# SIRT1 regulates MAPK pathways in vitiligo skin: insight into the molecular pathways of cell survival

**DOI:** 10.1111/jcmm.12206

**Published:** 2014-01-10

**Authors:** Matteo Becatti, Claudia Fiorillo, Victoria Barygina, Cristina Cecchi, Torello Lotti, Francesca Prignano, Agrippino Silvestro, Paolo Nassi, Niccolò Taddei

**Affiliations:** a Department of Biochemical Sciences, University of FlorenceFlorence, Italy; b Dermatology and Venereology Division, Guglielmo Marconi UniversityRoma, Italy; c Department of Critical Care Medicine, Dermatology Division, University of FlorenceFlorence, Italy

**Keywords:** SIRT1, Akt, MAPK, vitiligo, oxidative stress

## Abstract

Vitiligo is an acquired and progressive hypomelanotic disease that manifests as circumscribed depigmented patches on the skin. The aetiology of vitiligo remains unclear, but recent experimental data underline the interactions between melanocytes and other typical skin cells, particularly keratinocytes. Our previous results indicate that keratinocytes from perilesional skin show the features of damaged cells. Sirtuins (silent mating type information regulation 2 homolog) 1, well-known modulators of lifespan in many species, have a role in gene repression, metabolic control, apoptosis and cell survival, DNA repair, development, inflammation, neuroprotection and healthy ageing. In the literature there is no evidence for SIRT1 signalling in vitiligo and its possible involvement in disease progression. Here, biopsies were taken from the perilesional skin of 16 patients suffering from non-segmental vitiligo and SIRT1 signalling was investigated in these cells. For the first time, a new SIRT1/Akt, also known as Protein Kinase B (PKB)/mitogen-activated protein kinase (MAPK) signalling has been revealed in vitiligo. SIRT1 regulates MAPK pathway *via* Akt-apoptosis signal-regulating kinase-1 and down-regulates pro-apoptotic molecules, leading to decreased oxidative stress and apoptotic cell death in perilesional vitiligo keratinocytes. We therefore propose SIRT1 activation as a novel way of protecting perilesional vitiligo keratinocytes from damage.

## Introduction

Vitiligo appears as an enigmatic spectrum of depigmenting disorders affecting melanocytes to a different extent [1]. The aetiology of vitiligo remains unclear, but oxidative stress and the accumulation of free radicals have been proposed as important pathogenic mechanisms [2,3]. Recent experimental data underline the interactions between melanocytes and other typical skin cells, particularly keratinocytes. Previous results from our laboratory indicate that keratinocytes from perilesional skin show significant biochemical alterations, such as increased production of reactive oxygen species (ROS), lipoperoxidation and mitochondrial alterations, while apoptotic markers are primarily observed in keratinocytes from perilesional skin rather than in lesional or healthy skin [4]. This led us to hypothesize that perilesional vitiligo skin may represent the substrate where melanocyte death is initiated, with a substantial role played by keratinocytes in the development of disease.

Resveratrol (Resv) is a polyphenolic antioxidant containing two phenyl rings separated by a methylene bridge that is produced naturally in red grapes, berries and peanuts. Resveratrol can reduce the risk of several diseases and plays an important role in inflammation and tumour suppression [5]. Recent research has demonstrated that Resv activates the silent information regulator 2 (Sir2) family, and that the protective effects of Resv are mostly dependent on SIRT1 [6].

The members of the mammalian Sir2 family, or sirtuins, a cluster of highly conserved proteins eliciting nicotinamide adenine dinucleotide (NAD)-dependent histone deacetylase activity, have been shown to target histone and non-histone substrates, including enzymes, transcription regulators, tumour suppressors, cell signalling proteins and DNA repair proteins [7].

The requirement of NAD^+^ for the catalytic activity of sirtuins suggests they may have evolved as sensors of cellular energy and redox status. Several lines of evidence indeed point to a role for sirtuins in the response to changes in NAD^+^, Nicotinamide adenine dinucleotide and/or nicotinamide concentrations, as brought about by metabolic pathways [8,9]. To date, seven human sirtuins have been identified and named SIRT1-7 [10]. All these possess an NAD^+^-dependent catalytic core domain, which can function as an NAD^+^-dependent deacetylase (DAC) and/or mono-ADP-ribosyl transferase.

SIRT1 is the most extensively studied human sirtuin: indeed, 12 substrates with possible roles in cell processes ranging from cell survival to apoptotic signalling have been identified.

In addition to histones, SIRT1 also deacetylates several other proteins and emerges as a regulator of cellular mechanisms involved in stress responses.

Expression of all seven sirtuins has been demonstrated in human epidermal and dermal cells [11]. Sirtuins are involved in cellular pathways related to skin structure and function including photoageing, inflammation, cancer and cutaneous infections [12]. Two key mediators of skin damage, UV radiation and H_2_O_2_, down-regulate SIRT1 expression in cultured skin keratinocytes [13]. Resv activates SIRT1 in these UV-exposed keratinocytes, resulting in a decrease in p53-mediated apoptosis [13,14]. Conversely, pre-treatment with SIRT1 inhibitors sirtinol or nicotinamide promotes UV-induced, p53-mediated apoptosis [13]. Additional research has demonstrated decreased SIRT1, 3 and 7 expression in UVB-irradiated adult donor skin samples and cultured skin [15]. Decreased sirtuin expression was not identified in chronologically, non-sun exposed aged skin; however, suggesting that these sirtuins may be potential targets to prevent photoageing but not chronological ageing [15]. Therefore, upregulation of SIRT1, 3 and/or 7 are potential therapeutic targets to improve skin ageing and appearance [15].

Signs of oxidative stress and apoptosis in keratinocytes from perilesional vitiligo skin were found in our previous studies. In particular, we have observed high levels of activated p38, NF-kB p65 subunit, Smac/DIABLO and p53 [16]. On the basis of these data, we decided to investigate the role of SIRT1 signalling in these cells.

In the literature there is no evidence for SIRT1 signalling in vitiligo and its possible involvement in disease progression. Here we investigated a possible protective role played by this protein in keratinocytes from perilesional vitiligo skin.

## Materials and methods

### Keratinocyte isolation and cell culture

Punch-biopsies of 6 mm were taken from lesional, perilesional and healthy skin of 16 patients affected by non-segmental vitiligo all of which had a similar clinical history with respect to lesion extension and duration of the disease. All biopsies were taken from sun-unexposed areas. Lesional, perilesional and healthy skin is defined as clinically affected skin, skin along the edge of the white patch and clinically unaffected, normally pigmented skin respectively. Only patients with stable vitiligo were included in this study. The use of stable case of vitiligo to obtain skin biopsies has several technical advantages in the setting up and maintenance of primary cell cultures. Furthermore, it has enabled us to achieve a higher degree of reproducibility in the experiments. None of the patients underwent any kind of treatment (neither systemic nor topical) 6 months before biopsies were obtained. None of the patients we selected had any autoimmune disease in addition to vitiligo, nor did they have auto-antibodies against any organs. The clinical information of each patient is summarized in Table 1. Written permission was obtained from all patients. The study was conducted in accordance with the Declaration of Helsinki and was approved by the local institutional review board. Keratinocytes were isolated according to a previously published method [2]. The expression of cytokeratins was evaluated at each passage *in vitro* by a positive staining to pan-cytokeratin antibody (data not shown) to assess the maintenance of the same immunophenotype. At the first passage, the absence of vimentin expression induced us to exclude the presence of any fibroblasts.

**Table 1:** Clinical data of vitiligo patients

Patients	Age	Clinical type of vitiligo	Age of onset/Stability of vitiligo (years)	Biopsy site
M1	43	NS generalized	19/7	Trunk
M2	51	NS generalized	6/8.5	Trunk
M3	47	NS generalized	11/10	Abdomen
M4	39	NS generalized	5/8	Pubis
M5	52	NS generalized	25/7	Abdomen
M6	45	NS localized	21/10	Trunk
M7	48	NS generalized	22/9	Trunk
M8	52	NS generalized	23/8	Abdomen
F1	44	NS generalized	31/8	Abdomen
F2	38	NS generalized	20/7.5	Trunk
F3	49	NS generalized	13/9	Trunk
F4	42	NS generalized	33/8	Pubis
F5	50	NS generalized	27/7	Abdomen
F6	46	NS localized	18/9.5	Trunk
F7	44	NS generalized	28/10	Abdomen
F8	51	NS generalized	12/7	Trunk

### Cell treatment

To investigate the effect of SIRT1 activation, keratinocytes from perilesional vitiligo skin were grown for 24 hrs in the presence of 20 μM Resv. To see whether the effects of Resv were because of its antioxidant activity only, experiments were also performed in the same cells treated with 15 μM Trolox, a concentration required to obtain the same antioxidant capacity of 20 μM Resv.

Trolox (6-hydroxy-2, 5, 7, 8-tetramethylchromane-2-carboxylic acid) is a water-soluble analogue of the free radical scavenger α-tocopherol. Trolox has advantages over α-tocopherol, which is lipid soluble because it can be incorporated in both the water and the lipid compartments of cells [17–18].

To check possible toxic effects of these compounds, untreated cells were also considered and maintained in complete culture medium for the same time. In another set of experiments, cells were treated with 10 μM SB203580 (p38 kinase inhibitor), 10 μM SP600125 [JNK (c-Jun N-terminal kinase) inhibitor], 10 μM PD98059 (MEK inhibitor) [20], 1 μM 6-Chloro-2,3,4,9-tetrahydro-1H-Carbazole-1-carboxamide (specific SIRT1 inhibitor) for 1 hr. All reagents were reconstituted in dimethyl sulfoxide following the manufacturer's instructions. All reagents were purchased from Sigma-Aldrich (Milan, Italy) and were of the highest purity available.

### Preparation of cell homogenates

Keratinocytes from perilesional vitiligo skin (1 × 10^6^) were washed twice with PBS, trypsinized, centrifuged and then resuspended in 100 ml of lysis buffer containing 1% Triton X-100, 20 mM Tris-HCl pH8, 137 mM NaCl, 10% (v/v) glycerol, 2 mM ethylenediaminetetraacetic acid (EDTA) and 6 M urea supplemented with 0.2 mM PMSF, as well as 10 mg/ml leupeptin and aprotinin. To obtain cell homogenates, samples, after three freeze–thaw cycles, were twice sonicated in ice for 5 sec. and then centrifuged at 14,000 × *g* for 10 min. at 4°C. The supernatant was then collected. The protein concentration was determined according to the Bradford method [21].

### Determination of cellular SIRT1 activity

Cellular SIRT1 activity was determined according to the method described by Fulco *et al*. [22] with some modifications. Cell extracts were obtained by using a lysis buffer (50 mM Tris–HCl pH 8, 125 mM NaCl, 1 mM DTT, 5 mM MgCl_2_, 1 mM EDTA, 10% glycerol and 0.1% NP40 supplemented with 1 mM PMSF and protease inhibitors mix) and SIRT1 activities were determined by SIRT1 Direct Fluorescent Screening Assay Kit (Cayman, Ann Arbor, MI, USA). A volume of 25 μl of Assay buffer (50 mM Tris–HCl, pH 8.0, containing 137 mM NaCl, 2.7 mM Kcl, and 1 mM MgCl2), 5 μl of extracts and 15 μl of substrate solution was added to all wells. The plate was incubated on a shaker for 45 min. A quantity of 50 μl of Stop/Development solution was added to each well and incubated for 30 min. at room temperature. The fluorescence of the plate was then determined with a Perkin-Elmer LS 55 luminescence spectrometer with an excitation wavelength of 360 nm and an emission wavelength of 460 nm.

### Western blot analysis of p-ASK1

To assess the levels of p-apoptosis signal-regulating kinase-1 (ASK1) equal amounts of total homogenate (50 μg) were diluted in Laemmli sample buffer, boiled for 5 min. and separated on pre-cast 4–12% SDS-PAGE gels (Criterion XT, Bio-Rad Laboratories, Milan, Italy). Proteins were blotted onto polyvinylidene difluoride (PVDF) Hybond membranes, which were then incubated overnight at 4°C with (rabbit) anti-p-ASK1 pSer83 antibody (GenWay Biotech Inc.; rabbit, San Diego, CA, USA) anti-ASK1 (Santa Cruz Biotechnology Inc., Dallas, TX, USA). After washing, membranes were incubated with peroxidase-conjugated secondary antibodies for 1 hr. Immunolabelled bands were detected with a SuperSignal West Dura (Pierce, Rockford, IL, USA) and quantified with the aforementioned software for image analysis.

### Immunoprecipitations and immunoblot analysis

For immunoprecipitation, whole cell lysate were pre-cleared with Protein A/G plus (Santa Cruz Biotechnology Inc.) for 30 min at 4°C. Beads were pelleted at 1000 × *g* for 30 s and pre-cleared supernatants were incubated with 15 μg of primary antibody-agarose conjugates at 4°C overnight on a rotator. When agarose or a gel conjugate was unavailable, lysates were incubated with anti-Akt antibody (Santa Cruz Biotechnology Inc.) for 2 hrs at 4°C and then overnight along with Protein A/G plus beads to collect the immune complexes. Beads were collected by centrifugation, washed several times with RIPA buffer, one wash with PBS, and resuspended in SDS-PAGE sample loading buffer. Immune complexes and 80 μg of proteins were resolved by SDS-PAGE. Proteins were blotted onto PVDF Hybond membranes, which were then incubated overnight at 4°C with (mouse) anti-Akt antibody (mouse) anti-pAkt (mouse) anti-Ac-lysine (Santa Cruz Biotechnology Inc.). After washing, membranes were incubated with peroxidase-conjugated secondary antibodies for 1 hr. Immunolabelled bands were detected with a SuperSignal West Dura (Pierce).

### Determination of intracellular ROS and mitochondrial superoxide

Keratinocytes from perilesional vitiligo skin were seeded on glass cover slips and loaded with the mitochondrial superoxide-specific fluorescent probe MitoSOX (3 μM) and H_2_DCFDA (2.5 μM; Invitrogen, Carlsbad, CA, USA) – dissolved in 0.1% DMSO and Pluronic acid F-127 (0.01% w/v) – which was added to cell culture media for 15 min. at 37°C. Cells were fixed in 2.0% buffered paraformaldehyde for 10 min. at room temperature and the H_2_DCFDA and MitoSOX fluorescence analysed with a Leica TCS SP5 confocal scanning microscope (Mannheim, Germany) equipped with an argon laser for fluorescence analysis. A series of optical sections (1024 × 1024 pixels) 1.0 μm in thickness was taken through the cell depth at intervals of 0.5 μm with a Leica 20× objective and then projected as a single composite image by superimposition. Mitochondrial superoxide and ROS generation were also monitored by flow cytometry: single-cell suspensions were incubated with MitoSOX (0.5 μM) and H_2_DCFDA (1 μM; Invitrogen) for 15 min. at 37°C and immediately analysed with a FACSCanto flow cytometer (Becton-Dickinson, San Jose, CA, USA).

### Total antioxidant capacity (TAC)

Intracellular TAC, which accounts for ROS scavengers, was measured in cell lysates by chemiluminescent assay with the photoprotein Pholasin (Abel Antioxidant Test Kit; Knight Scientific Limited, Plymouth, UK), following the manufacturer's instructions. Protein content in the soluble fraction was measured with the Bradford method [21] and results calculated with an L-ascorbic acidbased standard curve.

### Evaluation of lipid peroxidation

To assess the rate of lipid peroxidation, isoprostane levels were measured in cell lysates with the 8-isoprostane EIA kit (Cayman Chemical Co.), following the manufacturer's instructions. Lipid peroxidation was also investigated by confocal scanning microscopy with BODIPY, a fluorescent probe that is intrinsically lipophilic and thus mimics the properties of natural lipids [23]. BODIPY 581/591 C11 acts as a fluorescent lipid peroxidation reporter that shifts its fluorescence from red to green in the presence of oxidizing agents. Briefly, cells were cultured on glass coverslips and loaded with dye by adding the fluorescent probe BODIPY, dissolved in 0.1% DMSO (5 mM final concentration), to the cell culture media for 30 min. at 37°C. The cells were fixed in 2.0% buffered paraformaldehyde for 10 min. at room temperature and the BODIPY fluorescence analysed (at an excitation wavelength of 581 nm) with a confocal Leica TCS SP5 scanning microscope equipped with an argon laser source for fluorescence measurements. A series of optical sections (1024 × 1024 pixels) 1.0 μm in thickness was taken through the cell depth at intervals of 0.5 μm with a Leica Plan Apo 63× oil immersion objective and then projected as a single composite image by superimposition. Moreover, lipid peroxidation was quantified by flow cytometry. Single-cell suspensions were washed twice with PBS and incubated, in the dark, for 30 min. at 37°C with BODIPY 581/591 (2 mM) in DMEM. After labelling, cells were washed and resuspended in PBS and analysed with a FACSCanto flow cytometer (Becton-Dickinson).

### Mitochondrial number

Mitochondrial number was determined with MitoTracker Deep Red 633 (Invitrogen), which was used to stain mitochondria, and confocal microscopy used to visualize individual mitochondria. Briefly, cells were seeded on glass cover slips and loaded with MitoTracker Deep Red 633 fluorescent probe (0.5 μM) – dissolved in 0.1% DMSO and Pluronic acid F-127 (0.01% w/v) – which was added to cell culture media for 20 min. at 37°C. Cells were fixed in 2.0% buffered paraformaldehyde for 10 min. at room temperature and red fluorescence analysed (at an excitation wavelength of 633 nm) with a Leica TCS SP5 confocal scanning microscope equipped with an argon laser source for fluorescence analysis. Mitochondrial number was also monitored by flow cytometry. Single-cell suspensions were incubated with MitoTracker Deep Red 633 (200 nM) for 20 min. at 37°C and immediately analysed by flow cytometry.

### Mitochondrial permeability transition pore opening

Mitochondrial permeability was analysed with the fluorescent calcein-AM probe, according to the method described by Petronilli *et al*. [24], albeit with minor modifications. Calcein-AM freely enters cells and emits fluorescence upon de-esterification. Co-loading of cells with cobalt chloride quenches cell fluorescence except in mitochondria. This is because cobalt cannot cross mitochondrial membranes (living cells). During mitochondrial permeability transition pore opening (mPTP), cobalt can enter mitochondria and is able to quench calcein fluorescence (apoptotic cells). Thus, decreased mitochondrial calcein fluorescence can be considered a measure of the extent of mPTP induction. Briefly, single-cell suspensions were incubated with the fluorescent probes calcein-AM (Invitrogen; 3 μM) and cobalt chloride (1 mM) for 20 min. at 37°C, washed twice with PBS and analysed with a FACSCanto flow cytometer (Becton-Dickinson).

### Mitochondrial membrane potential

Mitochondrial membrane potential was assessed with tetramethylrhodamine, methyl ester, perchlorate (TMRM). TMRM is a lipophilic potentiometric dye that partitions between the mitochondria and cytosol in proportion to Δψ by virtue of its positive charge. At low concentrations, the fluorescence intensity is a simple function of dye concentration, which is in turn a direct function of mitochondrial potential. Therefore, the accumulation of dye in mitochondria and the intensity of the signal is a direct function of mitochondrial potential. For confocal microscope analysis, cells were cultured on glass cover slips and loaded with dye by adding the fluorescent probe TMRM, dissolved in 0.1% DMSO (100 nM final concentration), to the cell culture media for 20 min. at 37°C. The cells were fixed in 2.0% buffered paraformaldehyde for 10 min. at room temperature and the TMRM fluorescence analysed (at an excitation wavelength of 543 nm) with a confocal Leica TCS SP5 scanning microscope equipped with a helium-neon laser source for fluorescence measurements. Mitochondrial membrane potential was also quantified by flow cytometry. Single-cell suspensions were washed twice with PBS and incubated, in the dark, for 20 min. at 37°C with TMRM (100 nM) in DMEM. After labelling, cells were washed and resuspended in PBS and analysed with a FACSCanto flow cytometer (Becton-Dickinson).

### Assessment of caspase activity by flow cytometry

Caspase-3 and caspase-9 activity was analysed by flow cytometry. In brief, single-cell suspensions were incubated with FAM-FLICA™ Caspases solution (Caspase FLICA kit FAM-DEVD-FMK) for 1 hr at 37°C, washed twice with PBS and analysed with a FACSCanto flow cytometer (Becton-Dickinson). In another set of experiments, designed to determine the relative importance of ERK, p38 and JNK signalling pathways, cells were treated with 10 μM SP600125 (specific JNK inhibitor), 10 μM PD98059 (MEK inhibitor) or 1 μM SIRT1 inhibitor for 1 hr prior to hypoxia.

### SIRT1 RNA interference (RNAi) experiments

**Figure 6:**
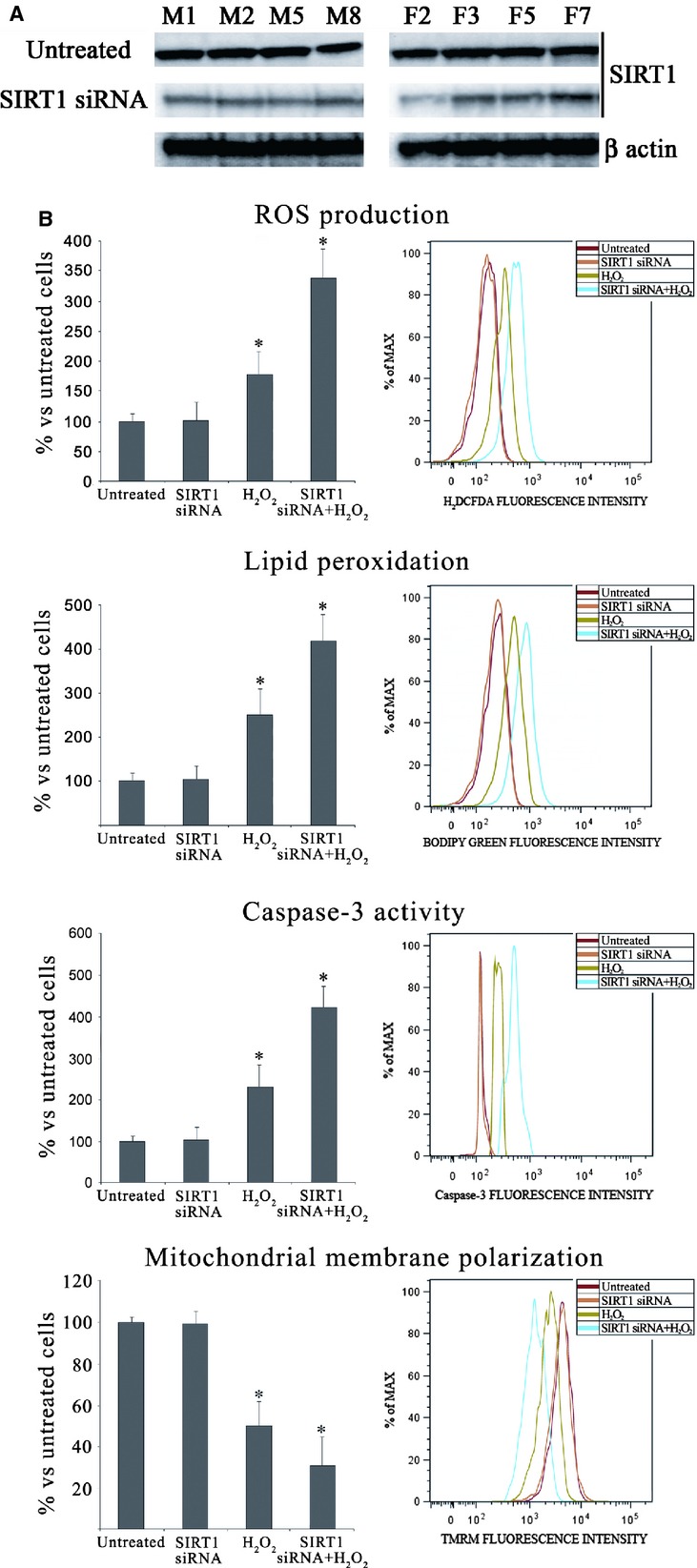
(A) In untreated keratinocytes from perilesional vitiligo skin and in keratinocytes from perilesional vitiligo skin after 48 hrs treatment of SIRT1 siRNA 100 nM, SIRT1 protein expression was determined by Western blot analysis. (B) To evaluate the possible involvement of SIRT1 in oxidative-mediated damage of vitiligo keratinocytes, untreated and SIRT1 RNAi-treated keratinocytes from healthy vitiligo skin were challenged for 3 hrs with 500 μM H_2_O_2_. Reactive oxygen species production, lipid peroxidation, caspase-3 activity and mitochondrial membrane polarization were measured by flow cytometry. These data confirm the key role of SIRT1 in vitiligo. The reported values (means ± SD) are representative of three independent experiments, each performed in triplicate. *Significant difference (*P* ≤ 0.05) *versus* untreated perilesional keratinocytes.

siRNA for SIRT1 (sc-40986) was purchased from Santa Cruz Biotechnology. Keratinocytes from healthy vitiligo skin (obtained from patients M1, M2, M5, M8 and F2, F3, F5, F7 as reported in Table 1) were cultured in complete medium that did not contain antibiotics for 2 days. Cells were seeded into a six-well plate and cultured to 60–70% confluence the day before the RNAi experiment when 8 μl of Lipofectamine™ LTX together 3 μl PLUS™ Reagent (Invitrogen, Indianapolis, IN, USA) was diluted in 90 μl of culture medium. Then, 12 μl SIRT1 siRNA was mixed with medium containing Lipofectamine together with PLUS reagent and incubated for 30 min. at room temperature for complex formation. Finally, the complex was added to the cells with the final SIRT1 siRNA concentration of 100 nM. SIRT1 protein expression was determined by Western blot after 48 hrs (Fig. 6A). To evaluate the possible involvement of SIRT1 in oxidative-mediated damage of vitiligo keratinocytes, untreated and SIRT1 RNAi-treated keratinocytes from healthy vitiligo skin were challenged for 3 hrs with 500 μM H_2_O_2_. ROS production, lipid peroxidation, caspase-3 activity and mitochondrial membrane polarization were measured by flow cytometry.

To shed light on the possible role of SIRT1 in oxidative-mediated damage in vitiligo keratinocytes, ROS production, lipid peroxidation, caspase-3 activity and mitochondrial membrane polarization were measured in keratinocytes from healthy vitiligo skin. These parameters were assessed in untreated and in SIRT1 siRNA-treated cells, challenged or not with H_2_O_2_ (500 μM for 3 hrs). In the absence of H_2_O_2_ challenge, the biochemical alterations displayed by SIRT1 siRNA-treated keratinocytes did not differ from unchallenged keratinocytes (Fig. 6B) in agreement with our recent findings obtained in a different cellular model [25]. The alterations of the above parameters were similar to those observed in keratinocytes from perilesional vitiligo skin when SIRT1 siRNA-treated keratinocytes were challenged with H_2_O_2_ (Fig. 6). This finding suggests that SIRT1 is a possible mediator of keratinocyte damage.

### Assessment of MAPK activity by flow cytometry

Flow cytometry is performed with a FACSCanto flow cytometer (Becton-Dickinson) and analysis is performed with FACSDiva software. Cells are fixed and permeabilized with the BD Cytofix/Cytoperm buffer (Becton-Dickinson) following the manufacturer's instructions. Anti-Phosphohyphen;SAPK/JNK (Thr183/Tyr185) (G9) Mouse mAb (PE Conjugate), anti-Phospho-p38 mitogen-activated protein kinase (MAPK; Thr180/Tyr182) (28B10) Mouse mAb (Alexa Fluor® 488 Conjugate) and anti-Phosphohyphen;p44/42 MAPK (Erk1/2) (T hr202/Tyr204) (D13.14.4E) XP® Rabbit mAb (Alexa Fluor® 488 Conjugate) were used at 1:50 dilution with 1 hr at room temperature incubation according to manufacturer's instructions.

### Statistical analysis

All data are expressed as mean ± SD. Comparisons between different groups were carried out with the one-way anova followed by Bonferroni *t*-test. A *P* < 0.05 was accepted as statistically significant.

## Results

### SIRT1 activity in keratinocytes from healthy, lesional and perilesional vitiligo skin

**Figure 1:**
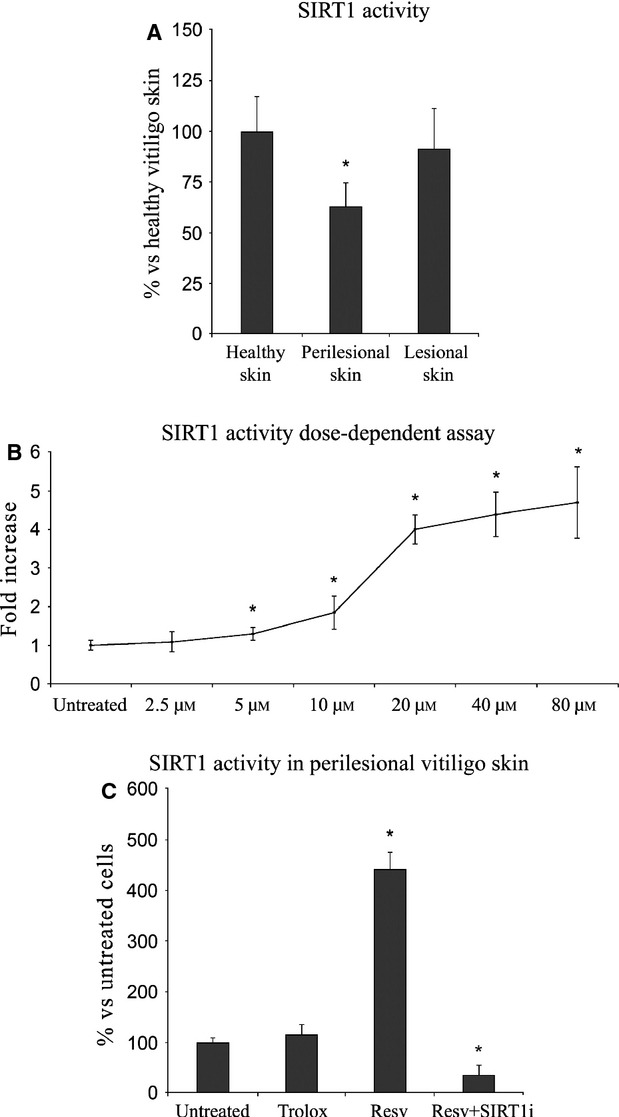
(A) SIRT1 activity in keratinocytes from healthy, perilesional and lesional vitiligo skin. *Significant difference (*P* ≤ 0.05) *versus* keratinocytes from healthy skins. (B) SIRT1 activity in keratinocytes from perilesional vitiligo skin after 24 hrs of incubation with different concentrations of Resveratrol (Resv). (C) SIRT1 activity in keratinocytes from perilesional vitiligo skin in the presence of 20 μM Resveratrol (Resv), 15 μM Trolox or 20 μM Resveratrol+1 μM SIRT1 inhibitor (Resv+SIRT1i). The reported values (means ± SD) are representative of four independent experiments, each performed in triplicate. *Significant difference (*P* ≤ 0.05) *versus* untreated perilesional keratinocytes.

As a preliminary experiment we evaluated the activity of SIRT1 in keratinocytes from healthy, lesional and perilesional skin (Fig. 1A). Compared to keratinocytes from healthy and lesional vitiligo skin, perilesional cells exhibited a marked decrease (−37% *versus* healthy skin, *P* < 0.001) in SIRT1 activity. Lesional cells showed only a mild decrease in SIRT1 activity respect to healthy skin from vitiligo patients (NS *versus* healthy skin).

Following this, all experiments were performed only in perilesional cells from vitiligo patients where we already reported [4,16] the presence of oxidative stress and apoptosis markers exclusively in keratinocytes from perilesional vitiligo skin.

### Dose-dependent effects of Resv on SIRT1 activity

As a preliminary experiment aimed at evaluating the effect of Resv on SIRT1 activity, a dose-dependent test was carried out in perilesional cells in the presence of increasing Resv concentrations (Fig. 1B). Keratinocytes from perilesional vitiligo skin were exposed to Resv concentrations ranging from 2.5 μM to 80 μM. Twenty-four hour treatment with 20 μM Resv stimulated a fourfold increase in SIRT1 activity and this Resv concentration was used for all other experiments.

### SIRT1 activity in keratinocytes from perilesional vitiligo skin

The effect of Resv, Trolox and Resv+SIRT1i on SIRT1 activity was investigated. As shown in Figure 1C, pre-treating with Resv up-regulated SIRT1 expression (fourfold increase *versus* untreated keratinocytes, *P* < 0.05); a pre-treatment with 15 μM Trolox did not induce a similar effect (NS *versus* untreated keratinocytes, *P* < 0.05). However, a significant decrease in SIRT1 activity was observed upon addition of a SIRT1 inhibitor to Resv-pre-treated cells (*P* < 0.05 *versus* untreated keratinocytes).

### Oxidative stress and SIRT1 activation in keratinocytes from perilesional vitiligo skin

**Figure 2:**
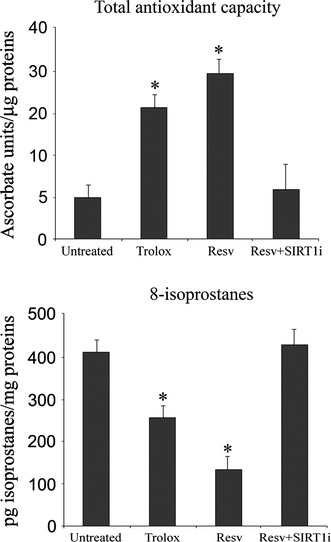
Evaluation of total antioxidant capacity (TAC) and 8-isoprostanes in keratinocytes from perilesional vitiligo skin in the presence of Resveratrol (Resv), Trolox or Resveratrol+SIRT1 inhibitor (Resv+SIRT1i). The reported values (means ± SD) are representative of four independent experiments, each performed in triplicate. *Significant difference (*P* ≤ 0.05) *versus* untreated perilesional keratinocytes.

**Figure 3:**
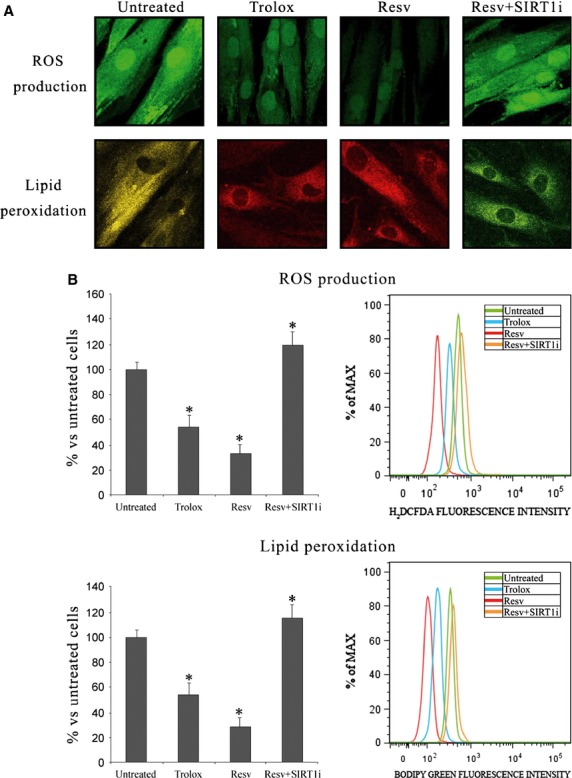
(A) Confocal microscope analysis and (B) flow cytometry analysis of reactive oxygen species production (63 ×  magnification) and lipoperoxidation (63 × magnification) in keratinocytes from perilesional vitiligo skin in the presence of Resveratrol (Resv), Trolox or Resveratrol+SIRT1 inhibitor (Resv+SIRT1i). The reported values (means ± SD) are representative of five independent experiments, each performed in triplicate. *Significant difference (*P* ≤ 0.05) *versus* untreated perilesional keratinocytes.

To investigate the effect of SIRT1 activation, keratinocytes from perilesional vitiligo skin were grown for 24 hrs in the presence of 20 μM Resv. To see whether the effects of Resv were merely because of its antioxidant activity, experiments were also performed in keratinocytes from perilesional vitiligo skin treated with 15 μM Trolox, the concentration which displayed *in vitro* the same antioxidant capacity of 20 μM Resv (data not shown). Keratinocytes treated with Resv and Trolox showed significantly higher TAC with respect to untreated cells; treatment with Resv caused a significant increase in cellular TAC, more so than Trolox (*P* < 0.05 *versus* Trolox-treated keratinocytes), suggesting that Resv-induced SIRT1 activation up-regulate different antioxidant pathways in response to oxidative stress compared to Trolox. The concentration of 8-isoprostanes was also much lower in Resv-treated cells compared to Trolox-treated cells (*P* < 0.05 *versus* Trolox-treated keratinocytes; Fig. 2). Lipoperoxidation was also monitored by flow cytometry and confocal microscopy analysis performed with the fluorescent probe BODIPY and similar results were obtained (Fig. 3).

**Figure 4:**
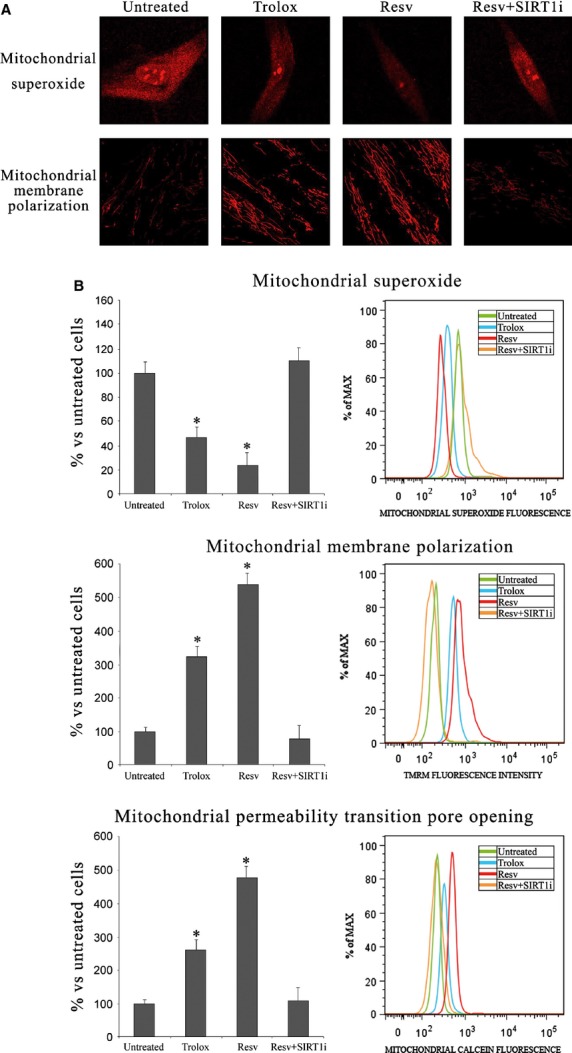
(A) Confocal microscope analysis of mitochondrial superoxide production and mitochondrial depolarization (63 ×  magnification) in keratinocytes from perilesional vitiligo skin in the presence of Resveratrol (Resv), Trolox or Resveratrol+SIRT1 inhibitor (Resv+SIRT1i). (B) Mitochondrial superoxide production, mitochondrial depolarization and mitochondrial permeability transition pore opening measured by flow cytometry in keratinocytes from perilesional vitiligo skin in the presence of Resveratrol (Resv), Trolox or Resveratrol+SIRT1 inhibitor (Resv+SIRT1i). The reported values (means ± SD) are representative of five independent experiments, each performed in triplicate. *Significant difference (*P* ≤ 0.05) *versus* untreated perilesional keratinocytes.

Reactive oxygen species production was investigated by flow cytometry and confocal microscopy analysis performed with the fluorescent probe H_2_DCFDA (Fig. 3). Resv-and Trolox-treated keratinocytes were characterized by less marked fluorescence, demonstrating a strong protective effect exerted by these compounds against ROS. Similar results were obtained evaluating mitochondrial superoxide production by flow cytometry and confocal microscope analysis (Fig. 4). Resv again proved to be more protective than Trolox.

### SIRT1 activation protects keratinocytes from perilesional vitiligo skin from mitochondrial damage and apoptosis

To ascertain whether SIRT1 activation can protect against apoptotic cell death, we analysed mitochondrial membrane polarization, the mitochondrial permeability transition pore opening and caspase activation. Figure 4 shows confocal microscope analysis of mitochondrial superoxide production, which appears strongly enhanced in untreated perilesional cells but not in antioxidant-treated cells. Mitochondrial membrane polarization, which was assessed by confocal microscopy and flow cytometry (Fig. 4), was found to be impaired in untreated perilesional keratinocytes. SIRT1 activation effectively restored mitochondrial membrane polarization. Trolox treatment showed similar effects, though to a lesser extent. To further confirm these data, mitochondrial permeability transition pore opening was analysed by flow cytometry (Fig. 4): Resv-induced SIRT1 activation again proved to be more protective compared to Trolox (Fig. 4). Treating keratinocytes from perilesional vitiligo skin with both Resv and a SIRT-1 inhibitor did not cause any significant change compared to untreated cells.

Mitochondrial number can influence experiments where mitochondrial function is investigated; we therefore counted the number of mitochondria in Resv-treated or non-treated keratinocytes from perilesional vitiligo skin to ensure that increased mitochondrial function was not because of a mere numeric increase in mitochondria. In our keratinocytes from perilesional vitiligo skin, no significant increase in mitochondrial number, compared to untreated cells, upon Resv treatment was evident (data not shown). Therefore, changes in mitochondrial function and cell viability are not because of changes in mitochondrial number.

**Figure 5:**
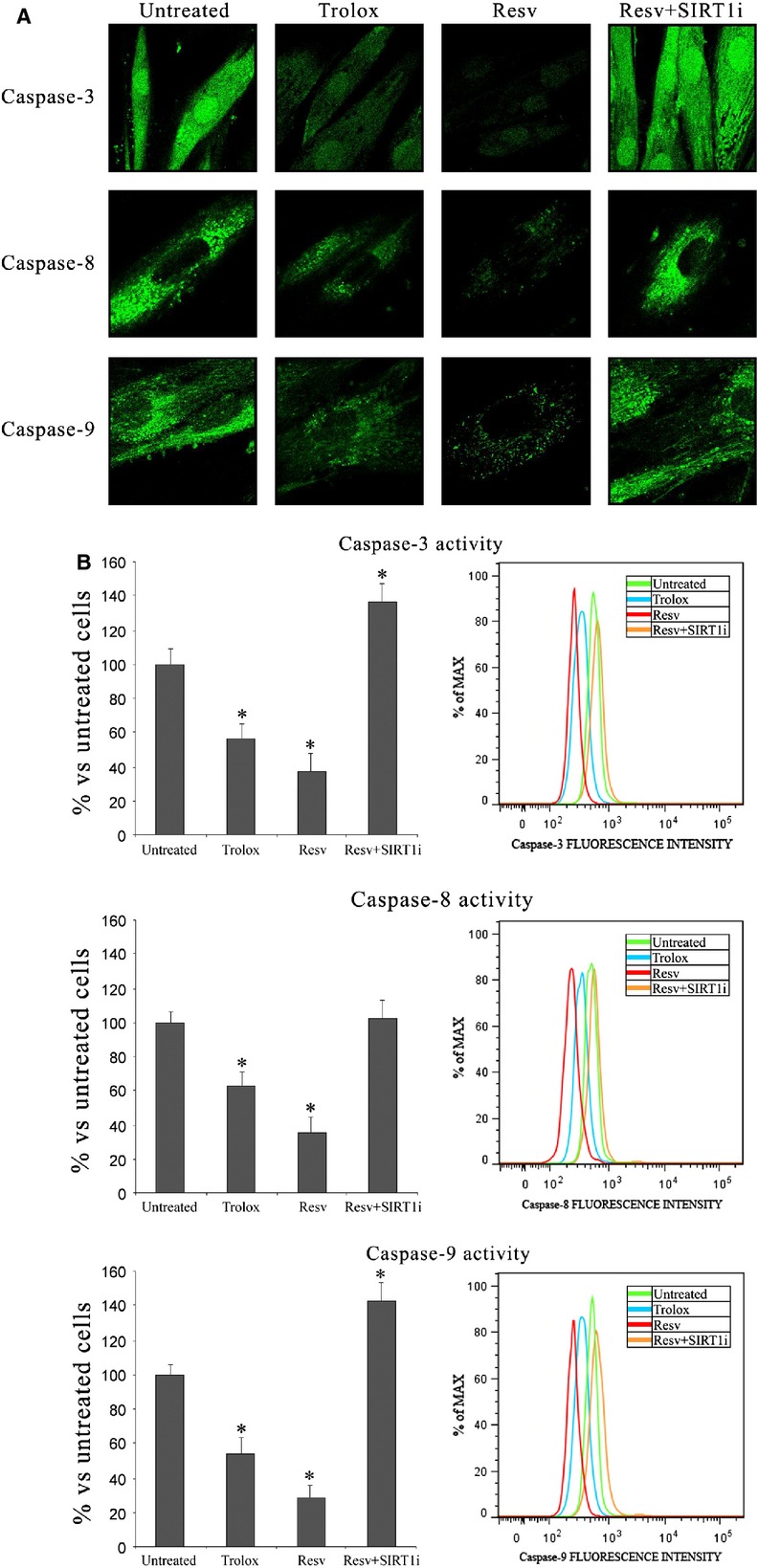
(A) Confocal microscope analysis and (B) flow cytometry analysis of caspases-3, 8 and 9 activation in keratinocytes from perilesional vitiligo skin in the presence of Resveratrol (Resv), Trolox or Resveratrol+SIRT1 inhibitor (Resv+SIRT1i). The reported values (means ± SD) are representative of five independent experiments, each performed in triplicate. *Significant difference (*P* ≤ 0.05) *versus* untreated perilesional keratinocytes.

We measured caspase-3,-8 and-9 activities to confirm that apoptosis occurs under our experimental conditions. This, together with the finding that Resv triggers SIRT1 activation, suggests that Resv may exert its anti-apoptotic effect in a SIRT1-dependent manner. As shown in our previous studies [4,16] a marked caspase-3,-8 and-9 activity, which plays a central role in apoptosis, was observed in keratinocytes from perilesional vitiligo skin and this increase was reversed by Resv pre-treatment. Treatment with Trolox, albeit to a lesser extent, significantly reduced apoptosis. In any case, a significant difference (*P* < 0.05) was observed between Resv and Trolox treatments, both for caspase-3,-8 and-9. A significant increase in caspase-3,-8 and-9 activity in the presence of SIRT-1 inhibitor (1 μM 6-Chloro-2,3,4,9-tetrahydro-1H-Carbazole-1-carboxamide) was also evident (Fig. 5).

### SIRT1 siRNA-treatment of keratinocytes from healthy vitiligo skin

In untreated keratinocytes from perilesional vitiligo skin and in keratinocytes from perilesional vitiligo skin after 48 hrs treatment of SIRT1 siRNA 100 nM, SIRT1 protein expression was determined by Western blot analysis to demonstrate the effective knockdown of SIRT-1 (Fig. 6A).

### MAPK pathways are affected by Resv-induced SIRT1 activation in keratinocytes from perilesional vitiligo skin

In our previous study, we demonstrated that keratinocytes from perilesional vitiligo skin showed signs of oxidative stress and apoptosis. In particular, we underlined the central role of MAPK pathways in inducing cell damage and apoptosis [4,16]. Here, we examined p38, ERK and JNK activation in response to Resv-induced SIRT1 activation.

**Figure 7:**
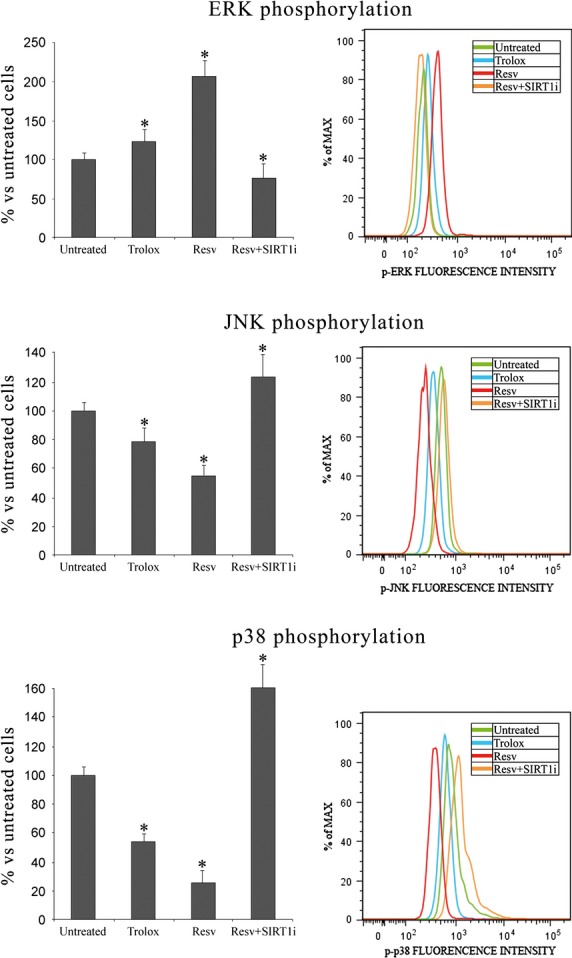
MAPK phosphorylation in keratinocytes from perilesional vitiligo skin in the presence of Resveratrol (Resv), Trolox or Resveratrol+SIRT1 inhibitor (Resv+ SIRT1i). The reported values (means ± SD) are representative of four independent experiments, each performed in triplicate. *Significant difference (*P* ≤ 0.05) *versus* untreated perilesional keratinocytes.

Figure 7 shows levels of ERK phosphorylation, whose anti-apoptotic effect is well documented. SIRT1 activation strongly increases ERK phosphorylation (+107% *versus* untreated keratinocytes, *P* < 0.05) whereas treatment with Trolox does not lead to a similar reduction in ERK phosphorylation. On the contrary, treatment with Resv and SIRT-1 inhibitor causes a significant reduction in ERK phosphorylation (−23% *versus* untreated keratinocytes, *P* < 0.05). In this study, we also investigated for the first time the role of JNK activation in keratinocytes from perilesional vitiligo skin. SIRT1 activation by Resv strongly reduced JNK phosphorylation (−46% *versus* untreated keratinocytes, *P* < 0.05), whereas Trolox treatment showed similar effects, even though to a lesser extent. Treatment with Resv and SIRT-1 inhibitor lead to an increase in JNK phosphorylation (+26% *versus* untreated keratinocytes, *P* < 0.05), demonstrating a role of SIRT1 in JNK pathways.

As previously demonstrated [16], p38 plays a prominent role in perilesional keratinocyte apoptosis *via* NF-kB and p53 activation. As already observed, SIRT1 activation significantly reduces p38 phosphorylation (−74% *versus* untreated keratinocytes, *P* < 0.05), whereas Trolox treatment did not lead to a similar reduction in p38 phosphorylation (−46% *versus* untreated keratinocytes, *P* < 0.05). When SIRT1 inhibitor was used with Resv, a dramatic increase in p38 phosphorylation (+60% *versus* untreated keratinocytes, *P* < 0.05) was shown. To further investigate the molecular pathways underlying the protective role of SIRT1 activation seen in keratinocytes from perilesional vitiligo skin, we analysed caspase-3 activity in the presence of specific MAPK inhibitors.

**Figure 8:**
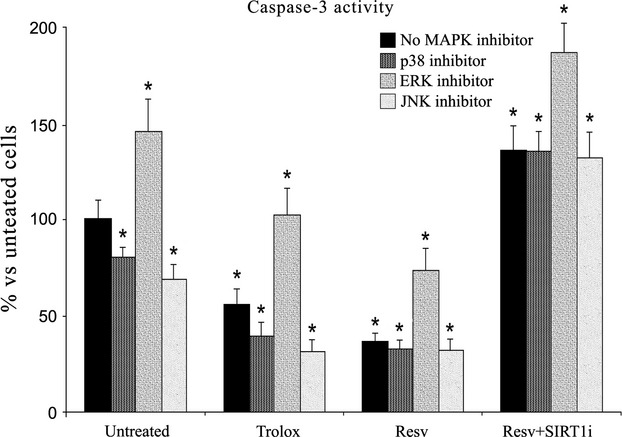
The caspase-3 activity measured with flow cytometry analysis in keratinocytes from perilesional vitiligo skin in the presence of specific MAPK inhibitors. The reported values (means ± SD) are representative of four independent experiments, each performed in triplicate. *Significant difference (*P* ≤ 0.05) *versus* untreated perilesional keratinocytes.

As shown in Figure 8, in the presence of p38 or JNK inhibitor (p38i or JNKi respectively), levels of apoptosis significantly decreased (*P* < 0.05 *versus* untreated keratinocytes), suggesting the involvement of these pathways in keratinocyte apoptosis. Interestingly, untreated keratinocytes displayed the greatest caspase-3 activity in the presence of ERK inhibitor, suggesting a prominent role for ERK in protection against apoptotic cell death.

When Resv was added along with these inhibitors, caspase-3 activity further decreased (*P* < 0.05 *versus* untreated keratinocytes). The presence of SIRT1 inhibitor together with p38i or JNKi triggered an increase in caspase-3 activity. Simultaneous treatment with the three inhibitors did not protect against cell death (data not shown).

### SIRT1 deacetylates Akt and inhibits ASK1 activation

**Figure 9:**
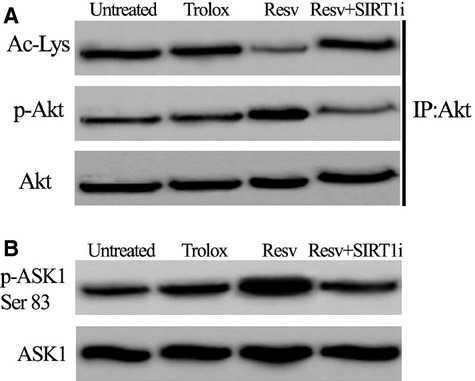
(A) Phosphorylation and acetylation status of Akt immunoprecipitated from phosphorylation in keratinocytes from perilesional vitiligo skin in the presence of Resveratrol (Resv), Trolox or Resveratrol+SIRT1 inhibitor (Resv+SIRT1i). (B) Western blot analysis of ASK1 phosphorylation at Ser83 in keratinocytes. The reported images are representative of four independent experiments.

It has been shown that SIRT1 activates Akt, which in turn can phosphorylate ASK1 at Ser-83 to maintain ASK1 in an inactive form [25]. Akt immunoprecipitates from perilesional keratinocytes indicated that phosphorylation of Akt was enhanced in Resv-treated cells, a finding that was associated with Akt deacetylation, indicating that SIRT1 deacetylates and activates Akt (Fig. 9A). When SIRT1 inhibitor was used, deacetylation and phosphorylation of Akt decreased to the levels observed in untreated keratinocytes, demonstrating that SIRT1 is necessary for Akt activation. Furthermore, a marked phosphorylation at Ser-83 of ASK1 (a specific Akt phosphorylation site) was present in Resv-treated keratinocytes indicating that Akt can inactivate ASK1 *via* SIRT1 pathway (Fig. 9B). When SIRT1 inhibitor was added, ASK1 phosphorylation level at Ser-83 greatly decreased.

## Discussion

Vitiligo is an autoimmune pigmentary disorder characterized by the loss of functional melanocytes from the involved epidermis. The aetiology of vitiligo, which is considered to be multifactorial, is still a matter of debate. Attention has been addressed to keratinocytes, which have been suggested to play a functional role in maintaining epidermal melanocytes [26].

Keratinocytes and melanocytes, being part of the epidermal-melanin unit, are connected with each other and to the basement membrane through adhesion molecules (E-cadherin, β-catenin and integrins) [27,28]. The detachment of melanocytes from basal membrane and their migration to the upper layer of epidermis could be because of a loss of dendricity of melanocytes or to an impairment of adhesion molecules [29]. In vitiligo melanocytes, an abnormal morphology of dendrites, possibly worsened by a high concentration of H_2_O_2_, has been suggested [29].

There is strong evidence that a melanogenic cytokine network able to regulate melanocyte function occurs between melanocytes and other types of skin cells, including keratinocytes and fibroblasts [30]. In fact, in co-culture systems constituted by keratinocytes and melanocytes, keratinocyte-derived factors induce melanocyte proliferation and differentiation.

Keratinocyte-derived stem cell factor (SCF) [31,32], endothelin-1 and granulocyte-macrophage colony-stimulating factor exert primary roles, in melanocyte dendritogenesis, proliferation and melanogenesis [33–36]. In addition, keratinocyte-derived basic fibroblastic growth factor (b-FGF) stimulates the proliferation of epidermal melanocytes [37]. The effects of the all above-mentioned cytokines and growth factors are mediated by the binding with specific receptors localized on the surface of melanocytes.

It has been demonstrated that protein and transcript levels of SCF are actually reduced in depigmented lesions although there is some debate about it [26,^38^–41]. This finding is also confirmed by the evidence that keratinocytes from vitiligo lesions appear to be more prone to apoptosis [42] and unable to generate sufficient levels of SCF to ensure melanocyte survival [39], compared to keratinocytes from normally pigmented skin. The reduced expression of SCF likely results from keratinocyte apoptosis and might be the cause of melanocyte death.

In active vitiligo, perilesional keratinocytes display a vacuolar appearance [43] suggesting that the impairment of keratinocyte function may be responsible for the loss of melanocytes *in vivo*, and may also explain the defective melanocyte growth seen *in vitro* [44]. Having said this, the molecular mechanisms that trigger keratinocyte apoptosis are still poorly understood.

Among the mechanisms that have been evoked in the modulation of cell stress response, the role of SIRT1 has been recently suggested [25,45] but its signalling and its possible involvement in vitiligo have never been explored. Here, for the first time, we shed light on a new SIRT1 signalling, which suggests a protective role in vitiligo keratinocytes.

We found that in keratinocytes from perilesional vitiligo skin oxidative stress was markedly pronounced, consistent with our previous results [4]. In contrast, keratinocytes treated with Resv and Trolox, used at an equivalent antioxidant capacity, displayed much lower levels of oxidative stress. Importantly, Resv displayed a greater protective effect than Trolox, an effect that is linked to SIRT1 activation. Also in Resv-treated mitochondria of perilesional vitiligo cells superoxide production, mitochondrial depolarization and impaired mPTP opening were inhibited to a greater extent with respect to Trolox treatment. In the presence of SIRT1 inhibitor all these effects were completely reversed. Hence, our results suggest that, in the presence of Resv, perilesional vitiligo keratinocytes are protected from cellular stress and concurrently show the activation of SIRT1. Resv has been shown to activate SIRT1, although it remains to be determined whether direct interaction, or the involvement of intermediates, is required [46]. However, it must be considered that some of the protective effects of Resv (*e.g*. its antioxidant properties) may be SIRT1-independent [47].

Our findings are in agreement with previous reports about a decreased protective action of Resv in response to SIRT1 knockdown [48] and with the lowering of mitochondrial ROS and cellular H_2_O_2_ production in cultured human coronary arterial endothelial cells (CAECs) following SIRT1 overexpression, thus recapitulating the effects of Resv [48]. Moreover, SIRT1-induced upregulation of antioxidants and downregulation of pro-apoptotic molecules, *via* FOXO activation and reduction in oxidative stress, has been shown to protect transgenic mice from IR-induced cardiac injury [49].

In our experimental model, following treatment with a SIRT1 inhibitor, Resv-induced protection against caspase activation was completely repressed, suggesting that SIRT1 mediates the protective effects of this compound. In this context it was recently shown that SIRT1 promotes expression of protective molecules, including MnSOD, Trx1 and Bcl-xL, while also down-regulating expression of pro-apoptotic effectors (*e.g*. Bax) [49]. Moreover, SIRT1 can inhibit apoptosis acting on p53 [45]. As p53 positively regulates Bax [50], it is possible that SIRT1 may partly exert its protective effects by deacetylating and inhibiting p53. Indeed, we have previously demonstrated a role for p53 in perilesional keratinocyte apoptosis and the relevance of this pathway in cell death [16].

Another important result of this study is the relationship between SIRT1 activation and MAPK signalling, an up-regulated pathway in response to increased ROS production [51], which has been yet shown to be markedly affected also in keratinocytes from perilesional vitiligo skin [16]. Here, with specific inhibitors, we showed that JNK and p38 kinases promote apoptosis, while ERK displays anti-apoptotic effects.

Activation of JNK and p38 was found in keratinocytes from perilesional vitiligo skin. Resv treatment, but not Trolox or SIRT-1 inhibitor, reduced phosphorylation to control levels. A recent study [13] found that siRNA-mediated SIRT1 knockdown in cultured keratinocytes promoted UV-induced JNK activation, and that this effect was reversed by treatment with Resv, suggesting that SIRT1 protects cells against UV-induced JNK activation. Previous reports conflict with our findings, and showed that JNK alters SIRT1 function and localization, while also stimulating its activation [52].

ERK has been shown to phosphorylate Thr202/Tyr204 in human fibroblasts in response to SIRT1 transfection and treatment with Resv [53]. How SIRT1 stimulates ERK phosphorylation remains to be determined: however, a recent study showed that MAPK signalling was suppressed by FK228, an HDAC inhibitor, which functions by up-regulating Rap1 expression [54]. We found that, in perilesional vitiligo cells, the p-ERK level was restored by Resv treatment and caspase-3 activity increased in the presence of ERK inhibitor, although this was reversed by SIRT1 activation, suggesting that SIRT1 protects against apoptosis. Indeed, our results indicate that ERK signalling partly underlies the protective effects of SIRT1.

To investigate the molecular mechanisms by which SIRT1 regulates MAPK pathways, we analysed the effects of SIRT1 on Akt (a serine/threonine kinase also known as protein kinase B, or PKB) and ASK1 signalling. While p38 and JNK mediate cell apoptosis and skin-damage processes, signals such as Akt negatively regulate this process [55–57]. Under normal physiological conditions, Akt is activated by growth factors, insulin, and DNA damage and is translocated from the cell membrane to its target genes, supporting the complex regulatory network [58]. Among its various cellular functions, there is extensive evidence that Akt plays a central role in regulating growth factor-mediated cell survival and blocking apoptosis [59]. Activated Akt relays its survival signals in a number of ways, including *via* phosphorylation and inactivation of pro-apoptotic factors such as ASK1 [60]. It has been shown that an active form of Akt blocks apoptosis *via* inhibition of AIF nuclear translocation [61] and that Akt directly phosphorylates human caspase-9, resulting in its inactivation [62]. Suppression of Akt leads to activation of the BCL-2 homology-3 domain-only proteins BIM and BAD, which in turn mediate the release of cytochrome *c*. Several lines of evidence have shown that Akt plays an essential role in protecting keratinocytes against UVB-induced apoptosis, by abolishing cytochrome *c* release and activation of caspases-3,-8 and-9 [57]. Furthermore, anti-apoptotic signals leading to the suppression of UVB-induced apoptosis by various factors have been shown to be mainly mediated through Akt activation [63]. In patients with vitiligo, Akt1 phosphorylation is reduced [64] and impaired PI3K/Akt activation is involved in keratinocyte apoptosis [65].

Many of the cellular processes regulated by Akt are also modulated by SIRT1: indeed, SIRT1 has been used to treat metabolic disorders characterized by aberrant Akt signalling [66].

In this study, we found that in perilesional vitiligo keratinocytes Akt phosphorylation levels increased in Resv-treated cells. This was associated with deacetylation of Akt, suggesting that SIRT1-mediated deacetylation and activation of Akt protects perilesional vitiligo keratinocytes from cell death. SIRT1-mediated deacetylation of Akt is required for its activation as it has been suggested recently by our and other groups[25,67], who showed that Akt phosphorylation decreased in the presence of a SIRT1 inhibitor, confirming that SIRT1 activity is required for Akt activation. Apoptosis signal-regulating kinase-1 is a MAPK kinase kinase (MAPKKK) family member [68], which is activated in response to oxidative stress, and other stimuli [69,70]. It is activated *via* distinct mechanisms and relays those signals to stress-activated MAP kinases, such as JNK and p38 [71,72]. Overexpression or hyperactivation of ASK1 causes mitochondria-dependent apoptosis, *via* cytochrome c release and activation of caspase-3/9 in various cell types [73,74]. Moreover, it is known that ASK1-mediated JNK activation phosphorylates Bcl-2, leading to a reduction in its anti-apoptotic activity [75]. However, the detailed molecular mechanisms that link mitochondria-dependent apoptosis and ASK1-p38/JNK activation remain unknown. In contrast, overexpression of ASK1 induces not only apoptosis but also cell differentiation and survival, depending on the cell type; for example, ASK1 induces neurite outgrowth in PC12 cells and keratinocyte differentiation *via* p38 pathway activation [76,77]. The mechanism that links ROS to the activation of p38 MAPK and JNK pathways involves regulation of the thioredoxin-ASK1 complex [(SH2)Trx-ASK1]. This complex inhibits the activity of ASK1 and, in turn, its activation of downstream p38 and JNK MAPK pathways [78]. In keratinocytes from perilesional vitiligo skin a decreased phosphorylation of ASK1 at Ser-83 and a concomitant marked increase in JNK and p38 phosphorylation were observed. We also found that ASK1 phosphorylation at Ser-83 returned to control levels following treatment with Resv. Reports suggest that H_2_O_2_ is responsible for decreased Akt and ASK1 (Ser-83) phosphorylation, as well as increased ASK1 phosphorylation at Thr-845. However, the H_2_O_2_ responsiveness of an ASK1 point mutant, ASK1S83A, was largely unaffected by Akt, suggesting a specific phosphorylation event in Akt-mediated inhibition [60].

To our knowledge, this is the first study to demonstrate the protective role of SIRT1 in vitiligo and its underlying mechanism. SIRT1 regulates MAPK signalling *via* Akt-ASK1 and down-regulates pro-apoptotic molecules, leading to decreased oxidative stress/apoptotic cell death in perilesional vitiligo keratinocytes. We therefore propose SIRT1 activation as a novel way of protecting perilesional vitiligo keratinocytes from damage. Pharmacological manipulation of sirtuin activity, which began with the use of Resv, has now extended to a variety of newer agents that appear to have greater specificity. Some of these agents are already in human clinical trials [79] and could be used as a therapeutical tool to protect against vitiligo progression.

## References

[1] Moretti S, Amato L, Bellandi S (2006). Focus on vitiligo: a generalized skin disorder. Eur J Inflamm.

[2] Passi S, Grandinetti M, Maggio F (1998). Epidermal oxidative stress in vitiligo. Pigment Cell Res.

[3] Schallreuter KU, Wood JM, Berger J (1991). Low catalase levels in the epidermis of patients with vitiligo. J Invest Dermatol.

[4] Prignano F, Pescitelli L, Becatti M (2009). Ultrastructural and functional alterations of mitochondria in perilesional vitiligo skin. J Dermatol Sci.

[5] Pervaiz S (2003). Resveratrol: from grapevines to mammalian biology. FASEB J.

[6] Monteserin-Garcia J, Al-Massadi O, Seoane LM (2013). SIRT1 inhibits the transcription factor CREB to regulate pituitary growth hormone synthesis. FASEB J.

[7] Bordone L, Guarente L (2005). Calorie restriction, SIRT1 and metabolism: understanding longevity. Nat Rev Mol Cell Biol.

[8] Gottlieb S, Esposito RE (1989). A new role for a yeast transcriptional silencer gene, SIR2, in regulation of recombination in ribosomal DNA. Cell.

[9] Aparicio OM, Billington BL, Gottschling DE (1991). Modifiers of position effect are shared between telomeric and silent mating-type loci in *S. cerevisiae*. Cell.

[10] Braunstein M, Rose AB, Holmes SG (1993). Transcriptional silencing in yeast is associated with reduced nucleosome acetylation. Genes Dev.

[11] Benavente CA, Schnell SA, Jacobson EL (2012). Effects of niacin restriction on sirtuin and PARP responses to photodamage in human skin. PLoS ONE.

[12] Serravallo M, Jagdeo J, Glick SA (2013). Sirtuins in dermatology: applications for future research and therapeutics. Arch Dermatol Res.

[13] Cao C, Lu S, Kivlin R (2009). SIRT1 confers protection against UVB-and H_2_O_2_-induced cell death *via* modulation of p53 and JNK in cultured skin keratinocytes. J Cell Mol Med.

[14] Chen M, Li J, Xiao W (2006). Protective effect of resveratrol against oxidative damage of UVA irradiated HaCaT cells. J Central South Univ.

[15] Lasserre C, D'Arcangelis A, Mildner M (2007). The effect of ultraviolet irradiation on sirtuin expression in human skin. J Invest Dermatol.

[16] Becatti M, Prignano F, Fiorillo C (2010). The involvement of Smac/DIABLO, p53, NF-kB, and MAPK pathways in apoptosis of keratinocytes from perilesional vitiligo skin: protective effects of curcumin and capsaicin. Antioxid Redox Signal.

[17] Distelmaier F, Visch HJ, Smeitink JA (2009). The antioxidant Trolox restores mitochondrial membrane potential and Ca^2^ ^+^ -stimulated ATP production in human complex I deficiency. J Mol Med.

[18] Raspor P, Plesnicar S, Gazdag Z (2005). Prevention of intracellular oxidation in yeast: the role of vitamin E analogue, Trolox (6-hydroxy-2,5,7,8-tetramethylkroman-2-carboxyl acid. Cell Biol Int.

[19] Poljsak B, Gazdag Z, Pesti M (2006). Role of the vitamin E model compound Trolox in the prevention of Cr(VI)-induced cellular damage. Toxicol Environ Chem.

[20] Bain J, Plater L, Elliott M (2007). The selectivity of protein kinase inhibitors: a further update. Biochem J.

[21] Bradford MM (1976). A rapid and sensitive method for the quantitation of microgram quantities of protein utilizing the principle of protein-dye binding. Anal Biochem.

[22] Fulco M, Cen Y, Zhao P (2008). Glucose restriction inhibits skeletal myoblast differentiation by activating SIRT1 through AMPK-mediated regulation of Nampt. Dev Cell.

[23] Drummen GPC, Gadella BM, Post JA (2004). Mass spectrometric characterization of the oxidation of the fluorescent lipid peroxidation reporter molecule C11-BODIPY581/591. Free Rad Biol Med.

[24] Petronilli V, Miotto G, Canton M (1999). Transient and longlasting openings of the mitochondrial permeability transition pore can be monitored directly in intact cells by changes in mitochondrial calcein fluorescence. Biophys J.

[25] Becatti M, Taddei N, Cecchi C (2012). SIRT1 modulates MAPK pathways in ischemic-reperfused cardiomyocytes. Cell Mol Life Sci.

[26] Moretti S, Spallanzani A, Amato L (2002). New insights into the pathogenesis of vitiligo: imbalance of epidermal cytokines at sites of lesions. Pigment Cell Res.

[27] Tang A, Eller MS, Hara M (1994). E-cadherin is the major mediator of human melanocyte adhesion to keratinocytes *in vitro*. J Cell Sci.

[28] Hara M, Yaar M, Tang A (1994). Role of integrins in melanocyte attachment and dendricity. J Cell Sci.

[29] Gauthier Y, Cario Andre M, Taïeb A (2003). A critical appraisal of vitiligo etiologic theories. Is melanocyte loss a melanocytorrhagy?. Pigment Cell Res.

[30] Imokawa G (2004). Autocrine and paracrine regulation of melanocytes in human skin and in pigmentary disorders. Pigment Cell Res.

[31] Halaban R, Tyrrell L, Longley J (1993). Pigmentation and proliferation of human melanocytes and the effects of melanocyte-stimulating hormone and ultraviolet B light. Ann NY Acad Sci.

[32] Grichnick JM, Burch JA, Burchette J (1998). The SCF/KIT pathway plays a critical role in the control of normal human melanocyte homeostasis. J Invest Dermatol.

[33] Hara M, Yaar M, Gilchrest BA (1995). Endothelin-1 of keratinocytes origin is a mediator of melanocyte dendricity. J Invest Dermatol.

[34] Imokawa G, Yada Y, Miyagishi M (1992). Endothelins secreted from human keratinocytes are intrinsic mitogens for human melanocytes. J Biol Chem.

[35] Yada Y, Higuchi K, Imokawa G (1991). Effects of endothelins on signal transduction and proliferation in human melanocytes. J Biol Chem.

[36] Imokawa G, Yada Y, Kimura M (1996). Granulocyte/macrophage colony-stimulating factor is an intrinsic keratinocyte-derived growth factor for human melanocytes in UVA-induced melanosis. Biochem J.

[37] Halaban R, Langdom R, Birchall N (1988). Basic fibroblastic growth factor from human keratinocytes is a natural mitogen for melanocytes. J Cell Biol.

[38] Moretti S, Spallanzani A, Amato L (2002). Vitiligo and epidermal microenvironment: possibile involvement of keratinocyte-derived cytokines. Arch Dermatol.

[39] Lee AY, Kim NH, Choi WI (2005). Less keratinocyte-derived factors related to more keratinocyte apoptosis in depigmented than normally pigmented suction-blisterd epidermis may cause passive melanocyte death in vitiligo. J Invest Dermatol.

[40] Bondanza S, Maurelli R, Paterna P (2007). Keratinocyte cultures from involved skin in vitiligo patients show an impaired *in vitro* behaviour. Pigment Cell Res.

[41] Kitamura R, Tsukamoto K, Harada K (2004). Mechanisms underlying the dysfunction of melanocytes in vitiligo epidermis: role of SCF/KIT protein interactions and its downstream effector. MITF-M. J Pathol.

[42] Lee AY, Youm YH, Kim NH (2004). Keratinocytes in the depigmented epidermis of vitiligo are more vulnerable to trauma (suction) the keratinocytes in the normally pigmented epidermis, resulting in their apoptosis. Br J Dermatol.

[43] Moellman G, Klein-Agarer S, Schollay DA (1982). Noncellular granular material and degeneration of keratinocytes in the normally pigmented epidermis of patients with vitiligo. J Invest Dermatol.

[44] Puri N, Mojamdar M, Ramajah A (1987). *In vitro* growth characteristics of melanocytes obtained from adult normal and vitiligo subjects. J Invest Dermatol.

[45] Alcendor RR, Kirshenbaum LA, Imai S (2004). Silent information regulator 2alpha, a longevity factor and class III histone deacetylase, is an essential endogenous apoptosis inhibitor in cardiac myocytes. Circ Res.

[46] Pacholec M, Bleasdale JE, Chrunyk B (2010). SRT1720, SRT2183, SRT1460, and resveratrol are not direct activators of SIRT1. J Biol Chem.

[47] Das S, Khan N, Mukherjee S (2008). Redox regulation of resveratrol-mediated switching of death signal into survival signal. Free Radic Biol Med.

[48] Ungvari Z, Labinskyy N, Mukhopadhyay P (2009). Resveratrol attenuates mitochondrial oxidative stress in coronary arterial endothelial cells. Am J Physiol Heart Circ Physiol.

[49] Hsu CP, Zhai P, Yamamoto T (2010). Silent information regulator 1 protects the heart from ischemia/reperfusion. Circulation.

[50] Miyashita T, Reed JC (1995). Tumor suppressor p53 is a direct transcriptional activator of the human bax gene. Cell.

[51] Torres M, Forman HJ (2003). Redox signaling and the MAP kinase pathways. BioFactors.

[52] Nasrin N, Kaushik VK, Fortier E (2009). JNK1 phosphorylates SIRT1 and promotes its enzymatic activity. PLoS ONE.

[53] Huang J, Gan Q, Han L (2008). SIRT1 overexpression antagonizes cellular senescence with activated ERK/S6k1 signaling in human diploid fibroblasts. PLoS ONE.

[54] Kobayashi Y, Ohtsuki M, Murakami T (2006). Histone deacetylase inhibitor FK228 suppresses the Ras-MAP kinase signaling pathway by upregulating Rap1 and induces apoptosis in malignant melanoma. Oncogene.

[55] Wan YS, Wang ZQ, Shao Y (2001). Ultraviolet irradiation activates PI 3-kinase/Akt survival pathway *via* EGF receptors in human skin *in vivo*. Int J Oncol.

[56] Umeda J, Sano S, Kogawa K (2003). *In vivo* cooperation between Bcl-xL and the phosphoinositide 3-kinase-Akt signaling pathway for the protection of epidermal keratinocytes from apoptosis. FASEB J.

[57] Wang HQ, Quan T, He T (2003). Epidermal growth factor receptor-dependent, NF-kappaB-independent activation of the phosphatidylinositol 3-kinase/Akt pathway inhibits ultraviolet irradiation-induced caspases-3,-8, and-9 in human keratinocytes. J Biol Chem.

[58] Vasudevan KM, Garraway LA (2010). AKT signaling in physiology and disease. Curr Top Microbiol Immunol.

[59] Hemmings BA (1997). Akt signaling–linking membrane events to life and death decisions. Science.

[60] Zhang R, Luo D, Miao R (2005). Hsp90-Akt phosphorylates ASK1 and inhibits ASK1-mediated apoptosis. Oncogene.

[61] Kim NH, Kim K, Park WS (2007). PKB/Akt inhibits ceramide-induced apoptosis in neuroblastoma cells by blocking apoptosis-inducing factor (AIF) translocation. J Cell Biochem.

[62] Cardone MH, Roy N, Stennicke HR (1998). Regulation of cell death protease caspase-9 by phosphorylation. Science.

[63] Decraene D, Agostinis P, Bouillon R (2002). Insulin-like growth factor-1-mediated Akt activation postpones the onset of ultraviolet B-induced apoptosis, providing more time for cyclobutane thymine dimer removal in primary human keratinocytes. J Biol Chem.

[64] Kim NH, Lee AY (2010). Reduced aquaporin3 expression and survival of keratinocytes in the depigmented epidermis of vitiligo. J Invest Dermatol.

[65] Kim NH, Jeon S, Lee HJ (2007). Impaired PI3K/Akt activation-mediated NF-kappaB inactivation under elevated TNF-alpha is more vulnerable to apoptosis in vitiliginous keratinocytes. J Invest Dermatol.

[66] Milne JC, Lambert PD, Schenk S (2007). Small molecule activators of SIRT1 as therapeutics for the treatment of type 2 diabetes. Nature.

[67] Sundaresan NR, Pillai VB, Wolfgeher D (2011). The deacetylase SIRT1 promotes membrane localization and activation of Akt and PDK1 during tumorigenesis and cardiac hypertrophy. Sci Signal.

[68] Ichijo H, Nishida E, Irie K (1997). Induction of apoptosis by ASK1, a mammalian MAPKKK that activates SAPK/JNK and p38 signaling pathways. Science.

[69] Takeda K, Matsuzawa A, Nishitoh H (2004). Involvement of ASK1 in Ca^2^ ^+^ -induced p38 MAP kinase activation. EMBO Rep.

[70] McDonald PH, Chow CW, Miller WE (2000). Beta-arrestin 2: a receptor-regulated MAPK scaffold for the activation of JNK3. Science.

[71] Chang H, Nishitoh H, Yang X (1998). Activation of apoptosis signal-regulating kinase 1 (ASK1) by the death adaptor Daxx. Science.

[72] Nishitoh H, Saitoh M, Mochida Y (1998). ASK1 is essential for JNK/SAPK activation by TRAF2. Mol Cell.

[73] Saitoh M, Nishitoh H, Fujii M (1998). Mammalian thioredoxin is a direct inhibitor of apoptosis signal-regulating kinase (ASK) 1. EMBO J.

[74] Hatai T, Matsuzawa A, Inoshita S (2000). Execution of apoptosis signal–regulating kinase 1 (ASK1)–induced apoptosis by the mitochondria-dependent caspase activation. J Biol Chem.

[75] Yamamoto K, Ichijo H, Korsmeyer SJ (1999). BCL-2 is phosphorylated and inactivated by an ASK1/Jun N-terminal protein kinase pathway normally activated at G (2)/M. Mol Cell Biol.

[76] Takeda K, Hatai T, Hamazaki TS (2000). Apoptosis signal–regulating kinase 1 (ASK1) induces neuronal differentiation and survival of PC12 cells. J Biol Chem.

[77] Sayama K, Hanakawa Y, Shirakata Y (2001). Apoptosis signal–regulating kinase 1 (ASK1) is an intracellular inducer of keratinocyte differentiation. J Biol Chem.

[78] Hsieh CC, Papaconstantinou J (2006). Thioredoxin-ASK1 complex levels regulate ROS-mediated p38 MAPK pathway activities in livers of aged and long-lived Snell dwarf mice. FASEB J.

[79] Elliott PJ, Jirousek M (2008). Sirtuins: novel targets for metabolic disease. Curr Opin Investig Drugs.

